# Downregulation of microRNA-200c-3p alleviates the aggravation of venous thromboembolism by targeting serpin family C member 1

**DOI:** 10.1080/21655979.2021.2005982

**Published:** 2021-11-29

**Authors:** Xiaorong Jian, Dehua Yang, Li Wang, Hongxiang Wang

**Affiliations:** a Department of Hematology, the Central Hospital of Wuhan, Tongji Medical College, Huazhong University of Science and Technology, Wuhan, Hubei, China; b Department of Pediatric Surgery, Union Hospital, Tongji Medical College, Huazhong University of Science and Technology, Wuhan430022, China

**Keywords:** Venous thromboembolism, miR-200c-3p, SERPINC1, antithrombin

## Abstract

Venous thromboembolism (VTE) is the third most prevalent cardiovascular complication. Increasing studies have demonstrated that some microRNAs (miRNAs) are aberrantly expressed in VTE and play crucial roles in mediating the development of VTE. Therefore, our study intends to explore the detailed function and molecular mechanism of miR-200c-3p in VTE progression. In our research, VTE rat models were first established via inferior vena cava (IVC) ligation and the time-dependent effects of IVC ligation on thrombus formation were discovered. The results of reverse transcription quantitative polymerase-chain reaction (RT-qPCR) and western blotting showed that serpin family C member 1 (SERPINC1) was downregulated in VTE rat models and showed an inverse correlation with thrombus load. MiRNA target prediction tools and luciferase reporter assay confirmed SERPINC1 as a target for miR-200c-3p. VTE rats were injected with miR-200c-3p inhibitor for 24 h to investigate whether miR-200c-3p knockdown influences thrombus formation *in vivo*. Histological examination through hematoxylin-eosin staining revealed that miR-200c-3p downregulation markedly inhibited the formation of thrombus in IVC of rats. Additionally, miR-200c-3p was upregulated while SERPINC1 was downregulated in serum and inferior vena cava of VTE rats as well as in plasma of patients with VTE. Linear regression analysis demonstrated that miR-200c-3p expression was negatively correlated to SERPINC1 expression in VTE rats and patients with VTE. Our study determines the previously unelucidated function of miR-200c-3p in VTE, which might provide a potential novel insight for the treatment of VTE.

## Graphical abstract

**Figure:**
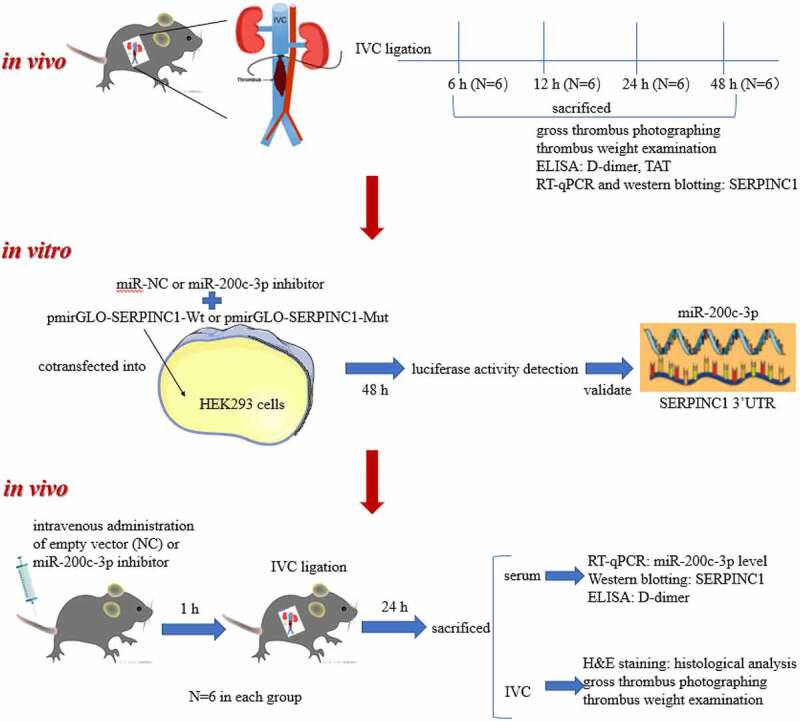

## Introduction

Venous thromboembolism (VTE) is the third most prevalent cardiovascular disorder with the incidence of 1–2 per 1000 individuals every year [1,2]. It may result in severe short-term or long-term complications such as post-thrombotic syndrome, chronic thromboembolic pulmonary hypertension, or even death [3–5]. The combination of human epidemiological data and genetically manipulated animal models indicates that various acquired or genetic risk factors are related to VTE, contributing to its multifactor complexity [6–8]. At present, epigenetics biomarkers are used for VTE diagnosis and prevention and the role of statins, a class of lipid-lowering epigenetic-based drugs as additional therapeutic agents in VTE is under investigation [9]. Therefore, it is crucial to determine the molecular pathways that control the blood clot development and are associated with the pathogenesis of VTE for performing targeted prevention and treatment.

Antithrombin (AT), as a main circulating inhibitor of blood coagulation proteases, belongs to the serine protease inhibitor (SERPIN) superfamily [10]. Serpin family C member 1 (SERPINC1), which is located on chromosome 1q25.1 comprising 6 introns and 7 exons, encodes AT in humans [11]. Congenital AT defect is an autosomal-dominant thrombotic disease [12], which is related to the high risk of VTE [13]. A previous study in experimental mouse models showed that the homozygous null deficiency in SERPINC1 led to serious thrombosis and bleeding, eventually causing embryonic death [14]. Nevertheless, the molecular mechanism of SERPINC1 in the development of venous thromboembolism deserves further exploration.

MicroRNAs (miRNAs) are non-coding RNAs that modulates gene expression via translational inhibition of multiple targets or mRNA degradation [15]. Each miRNA may target multiple mRNAs at the same time and suppress their expression to alter biological networks [16,17]. MiRNAs are mainly located in cells; however, miRNAs can also be detected outside the cell in various body fluids such as serum and plasma [18]. As one of the most abundant nucleic acids, miRNAs can be packaged and transported to the recipient cells by extracellular vesicles [19,20]. Existing experimental reports emphasize the pivotal function of miRNAs in cardiovascular physiology, biology, and disease [21]. It was reported that several miRNAs including miR-146, miR-21, miR-126, and miR-155 modulate pathogenic signaling in the development of coronary artery disease, atherosclerosis, neointimal lesion formation, and vascular inflammation [22–24]. Furthermore, increasing evidence from animal models and epidemiological studies suggests that some miRNAs are differentially expressed in VTE [25,26]. Recently, miR-200c-3p was demonstrated to be downregulated in hibernating black bear plasma to prevent venous thromboembolism during prolonged immobility [27]. However, to our knowledge, the specific role and regulatory mechanism of miR-200c-3p in the pathogenesis of VTE have not yet been elucidated.

In this report, we aimed to explore the role and molecular mechanism of miR-200c-3p in VTE development. We proposed a hypothesis that miR-200c-3p downregulation suppresses the formation of thrombus in VTE by upregulating SERPINC1. Our study may provide a potential novel insight in treatment of VTE.

## Materials and methods

### Patients

Patients with unprovoked VTE were recruited from the Central Hospital of Wuhan (Hubei, China). Subjects aging 20–80 years, who suffered from an unprovoked VTE without recurrence 1–5 years before this study and whose anticoagulant and antiplatelet treatment was stopped at least 3 months before this study, were considered eligible for this study. Subjects with cancer or other serious life-threatening medical conditions, present or previous cardiovascular diseases such as recurrent venous thrombosis, myocardial infarction and stroke, diabetes mellitus, angina pectoris and current abuse of drugs or alcohol were excluded from the study.

Forty-seven VTE patients who fulfilled the criteria and responded positively to an invitation letter were recruited for this study. All patients were diagnosed by Color duplex ultrasound [28]. Fourteen healthy volunteers who matched for age and sex as well as underwent the same screening visit as the VTE patients were chosen as controls. The demographic characteristics for VTE patients and healthy controls were listed in Table 1. Prior to the study, all subjects were fully informed of the purpose, nature of and risks of participation, and signed written informed consent. The study was approved by the ethics review committee of the Central Hospital of Wuhan.

**Table 1.** Clinical data of the study population

Characteristic	Controls (n = 14)	Unprovoked VTE (n = 47)	*p*-value
Age (years, mean±SD)	58.44 ± 10.86	59.50 ± 9.27	0.74
Male (%)	4(28.57)	29(61.70)	0.029
Hypertension (%)	9(64.29)	26(55.32)	0.552
Diabetes Mellitus (%)	2(14.29)	25(53.19)	0.010
Lipidemia (%)	2(14.29)	7(14.89)	0.955
Smoking (%)	9(64.29)	24(51.06)	0.384

### Human blood sample collection and isolation

The collection and isolation of human blood samples were previously described [29]. Briefly, participants were fasted overnight for 12 h, and blood was drawn from an antecubital vein and collected into 5-mL Vacutainer tubes (Becton Dickinson, Meylan Cedex, France) containing EDTA as an anticoagulant. Platelet-poor plasma was obtained by centrifugation at 3000 g for 10 min at 22°C, after which the supernatant was transferred into cryovials (Greiner Laboratechnik, Nurtringen, Germany) in 1-mL aliquots and stored at −80°C until further analysis.

### Cell culture

Human kidney embryonic cells (HEK293) were bought from American Type Culture Collection (ATCC, Manassas, VA). HEK293 cells were seeded in 24-well plates in Dulbecco’s modified Eagle medium (DMEM; Thermo Fisher Scientific) containing 10% fetal bovine serum (FBS; Gibco) and maintained in 5% CO_2_ incubator at 37°C for later luciferase reporter assays [30].

### Animal studies

Animal experiments were conducted following the Institute for Laboratory Animal Research guidelines. Age matched 250–300 g Sprague-Dawley rats (Beijing Vital River Laboratory Animal Technology Co., Ltd., Beijing, China) were used. As previously described, we established stasis-induced VTE animal model [31,32]. In brief, rats were placed in the supine position after being anesthetized with ketamine (100 mg/kg) and xylazine (20 mg/kg). After the midline laparotomy, the inferior vena cava (IVC) was carefully separated from the surrounding tissues and then ligated below the renal veins along with ligation of side branches. After 6 h, 12 h, 24 h, and 48 h of ligation, rats were euthanized and the IVC was dissected. Thrombus was extracted, whose weight was measured in milligrams. The IVC with thrombus was fixed in formalin and then stained with hematoxylin and eosin.

### Reverse transcription quantitative polymerase chain reaction (RT-qPCR)

Total RNA was isolated from rat IVC tissues, rat serum, or human plasma using TRIzol reagent (Invitrogen). Media and intimal RNA was isolated from IVC as previously described [33]. In brief, IVC was cut out and transferred to a medium supplemented with ice-cold phosphate buffer saline (PBS). The tip of an insulin syringe needle was carefully inserted into one end of the IVC to facilitate rapid wash of QIAzol lysis buffer, and intima eluate was collected. The IVC leftover (medium + adventitia) was washed with PBS and snap-frozen in liquid nitrogen for later extraction of total RNA. RNA extraction from rat serum or human plasma was described previously [34]. Briefly, the miRNA was extracted from 5 ml of the plasma or serum using an mirVana™ miRNA Isolation kit (Ambion, Austin, TX, USA). The quantity and quality of the obtained RNA was measured using a NanoDrop ND-1000 spectrophotometer (NanoDrop, Wilmington, DE, USA). For mRNA RT-qPCR, 1 μg of total RNA was reverse transcribed into complementary DNA (cDNA) using the Quantitect Reverse Transcription Kit (Qiagen). RT-qPCR was performed using a CFX 96 connect real-time PCR system (BioRad). MiRNA RT-qPCR was performed using Stem-loop RT-qPCR method [35]. Total RNA (500 ng) was reverse transcribed using miRNA-specific RT primers via SuperScript-III reverse transcriptase (Invitrogen). The PCR reaction was performed at 95°C for 5 min, followed by 45 cycles at 94°C for 15 sec, 58°C for 30 sec, and 72°C for 30 sec. Each PCR was repeated in triplicate. The primer sequences were displayed in Table 2. Finally, the relative expression level of miRNA and mRNA was normalized against that of U6 snRNA and glyceraldehyde-3-phosphate dehydrogenase (GAPDH), and calculated using the 2^−ΔΔCt^ method [36].

**Table 2.** Primer sequences used for RT-qPCR

Gene	Species	Primer sequences
SERPINC1	Mouse	Forward: 5′-GGTGAGAGGAAGCTTTGTC-3′
		Reverse: 5′-CTATGCAGATGTCGTCCAC-3′
GADPH	Mouse	Forward: 5′-ACTCTTCCACCTTCGATGC-3′
		Reverse: 5′-CCGTATTCATTGTCATACCAGG-3′
miR-140-5p	Mouse	Forward: 5′-CGTATCCAGTGCAATTGCCG-3′
		Reverse: 5′-GTCGTATCCAGTGCGTGCG-3′
miR-200b-3p	Mouse	Forward: 5′-CACATCCACCTCCTCCACATC-3′
		Reverse: 5′-AATGCGGCCGCAACTCAATCAACATCACCAT-3′
miR-200c-3p	Mouse	Forward: 5′-AGCGGTAATACTGCCGGGTA-3′
		Reverse: 5′-GTGCAGGGTCCGAGGT-3′
miR-429	Mouse	Forward: 5′-ATACTGTCTGGTAATGCCG-3′
		Reverse: 5′-GAACATGTCTGCGTATCTC-3′
miR-203-3p	Mouse	Forward: 5′-GGCGGGCTGAAATGTTTAGGA-3′
		Reverse: 5′-GTGCAGGGTCCGAGGTATTC-3′
U6	Mouse	Forward: 5′-CTCGCTTCGGCAGCACATATACT-3′
		Reverse: 5′-CGCTTCACGAATTTGCGTGT-3′
miR-200c-3p	Human	Forward: 5′-AGGGCTAATACTGCCGGGTAA-3′
		Reverse: 5′-CAGTGCAGGGTCCGAGGTAT-3′
U6	Human	Forward: 5′-ATACAGAGAAAGTTAGCACGG-3′
		Reverse: 5′-GGAATGCTTCAAAGAGTTGTG-3′

### Luciferase reporter assay

To investigate the interaction between SERPINC1 and miR-200c-3p, wide type or mutant 3′-UTR of SERPINC1 was cloned into the firefly luciferase gene reporter vector pmiRGLO (Promega, Madison, WI, USA). The plasmid was synthesized by Invitrogen. The pmirGLO-SERPINC1-Wt or pmirGLO-SERPINC1-Mut was co-transfected with miR-NC or miR-200c-3p inhibitor (RiboBio, Guangzhou, China) into HEK293 cells using Lipofectamine 3000 (Invitrogen). The blank cells were regarded as control cells. Forty-eight h post transfection, cells were harvested. Luciferase assay was performed using the Dual Luciferase Reporter Assay System (Promega) under the protocol of manufacturer. The relative luciferase expression equaled the expression of the Renilla luciferase divided by the expression of the firefly luciferase [37]. Transfection was repeated for at least 3 times.

### In vivo *miRNA delivery*

Briefly, mirVana miRNA inhibitor (Life Technologies) was suspended in invivofectamine 2.0 (Invitrogen) following the manufacturer’s instructions to form nanoparticles suitable for *in vivo* applications. Each rat (n = 6/group) was injected tail vein with 200 μl of mixtures containing empty vector (NC) or miR-200c-3p inhibitor (1 nmol-10 nmol) 1 h before IVC ligation as previously described [33].

### Histological examination

IVC containing thrombus was extracted, fixed with 4% paraformaldehyde and embedded in paraffin. Then, 5 μm of serial sections were sliced for the analysis of thrombus formation. Sections were stained with hematoxylin and eosin according to standard procedures. An inverted microscope (Motic) was used to capture histological images [38].

### Western blotting

Proteins were extracted from IVC tissues using radioimmunoprecipitation assay lysis buffer containing complete protease inhibitor cocktail. Then, equal amounts of proteins were loaded on 10% sodium dodecyl sulfate polyacrylamide gel electrophoresis gel for separation, and transferred onto polyvinylidene fluoride membranes (Millipore, Billerica, MA). After blocked with nonfat milk for 1 h, the membranes were bred overnight at 4°C with proper dilutions of primary antibodies: anti-SERPINC1 (ab126598; Abcam, Shanghai, China) and anti-GAPDH (ab125247). The membranes were further incubated with horseradish peroxidase-conjugated secondary antibodies after being washed using Tris-Buffered Saline Tween-20 (TBST) solution. Bands were detected using chemiluminescence substrate (Sigma) and ImageJ software (NIH) was applied to quantify the intensity of bands [39].

### Enzyme-linked immunosorbent assay (ELISA)

Circulating levels of rat serum D-dimer and TAT were assessed by the commercially available rat D-dimer and TAT ELISA kits (Sincere Biotech) following the manufacturer’s protocols [40].

### Statistical analysis

Data are presented as the mean ± standard deviation and were analyzed with GraphPad Prism 6 software (GraphPad Software, Inc., USA). P-values were obtained using the Student’s *t*-test to compare two groups or one-way analysis of variance (ANOVA) with Tukey’s *post hoc* test to compare multiple groups, and denoted as: ***p < 0.001, **p < 0.01 and *p < 0.05. Linear regression analysis was used for assessing the correlation between parameters. Each experiment was repeated in triplicate.

## Results

In this study, we aimed to investigate the function of miR-200c-3p in the development of VTE and the underlying molecular mechanisms. We hypothesized that miR-200c-3p knockdown alleviated thrombus formation by targeting SERPINC1, the gene for antithrombin. We initially established VTE rat models via IVC ligation and discovered that SERPINC1 was significantly downregulated in VTE rat model. Through TargetScan database and luciferase reporter assay, the binding between miR-200c-3p and SERPINC1 3ʹUTR was validated. Afterward, VTE rats were injected with miR-200c-3p inhibitor. After 24 h, rats were sacrificed for histological examination of IVC containing thrombus by HE staining. The thrombus weight and the expression of D-dimer, the biomarker for thrombus formation, were also detected 24 h after downregulating miR-200c-3p. Finally, the expression of miR-200c-3p and SERPINC1 as well as the correlation between them in VTE rats and VTE patients were examined. The findings demonstrated that miR-200c-3p knockdown alleviates the aggravation of VTE by targeting SERPINC1.

### SERPINC1 is downregulated in VTE rat models

**Figure 1.**
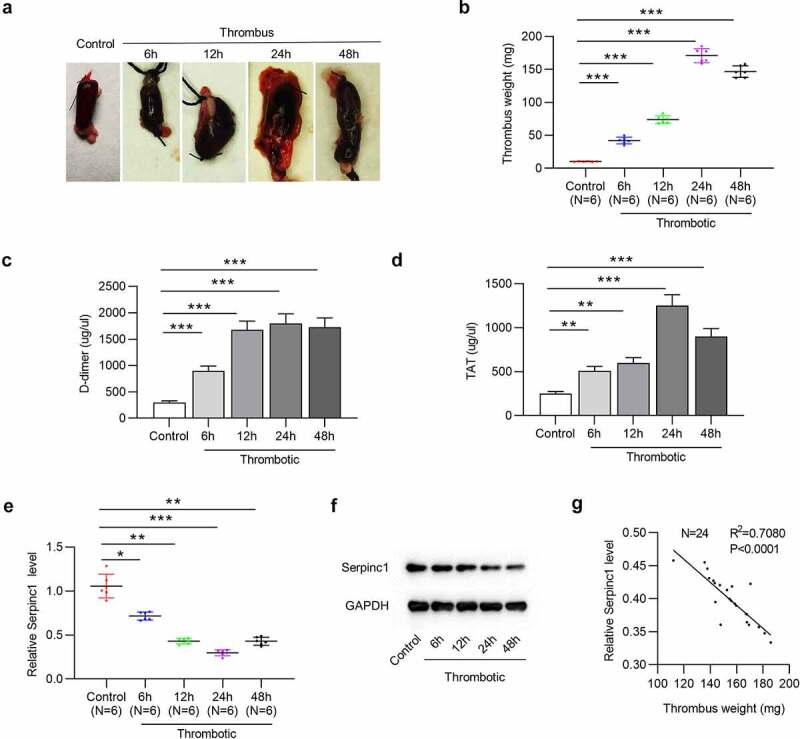
SERPINC1 is downregulated in serum of VTE rats

To investigate the specific molecular mechanism underlying VTE, we mimicked the characteristics of VTE by establishing VTE rat models through IVC ligation. First, gross thrombus and thrombus weight at different time points (6 h, 12 h, 24 h and 48 h post-IVC ligation) were subjected to an in-situ inspection, which exhibited that the ligation contributed to the development of thrombi inside IVC in a time-dependent manner (Figure 1a-b). Moreover, the levels of D-dimer and TAT, biomarkers for thrombus formation, in serum were discovered to be up surged till 24 h post ligation (Figure 1c-d). These findings suggested that VTE rat models were established. The protein SERPINC1 has been reported to be closely associated with severe thrombosis [14]. Therefore, we then measured SERPINC1 level in serum of VTE rats at different time points, and discovered that SERPINC1 mRNA and protein levels were gradually reduced with the thrombus progression at 6, 12, 24 and 48 h, as shown by RT-qPCR and western blotting (Figure 1e-f). Furthermore, linear regression analysis demonstrated a significant inverse correlation between thrombus weight and SERPINC1 expression in VTE rats at stipulated time points (Figure 1g).

(a) Representative images for gross IVC with thrombus obtained from rats at indicated time point (6 h, 12 h, 24 h, and 48 h post IVC ligation). (b) Thrombus weight, (c) D-dimer serum level and (d) TAT serum level from control and thrombotic rats at indicated time points. n = 6. (e-f) SERPINC1 mRNA level and protein level in control or thrombotic group at indicated time points was examined using RT-qPCR and western blotting, respectively. n = 6. (g) Scatter plot analysis of the correlation between SERPINC1 level and thrombus weight of rats in the thrombotic group. n = 24. *p < 0.05, **p < 0.01, ***p < 0.001.

### SERPINC1 3ʹUTR is targeted by miR-200c-3p

**Figure 2.**
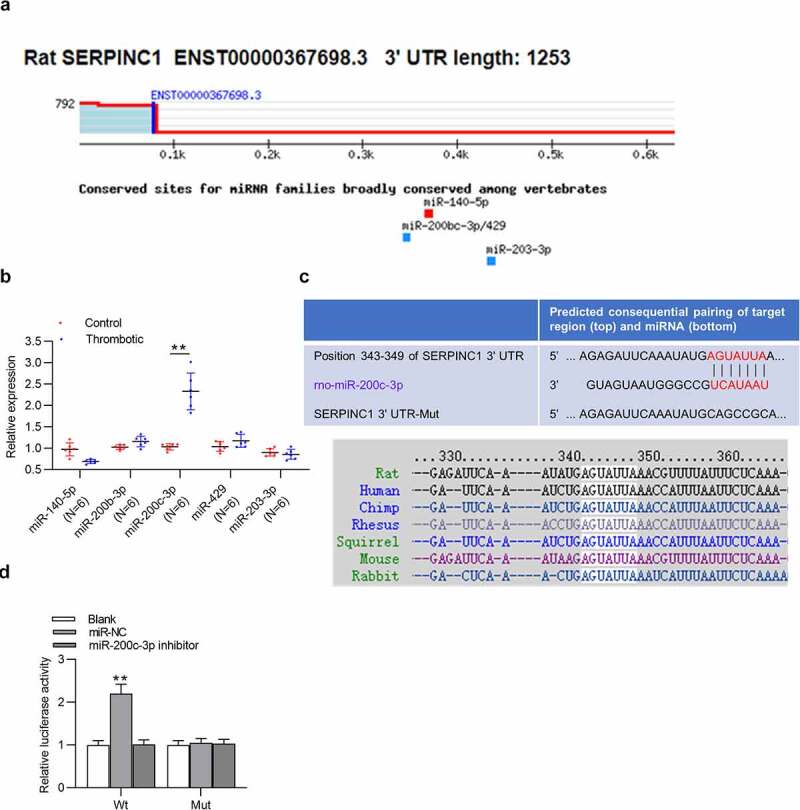
SERPINC1 is targeted by miR-200c-3p

Through TargetScan database, we found five highly conserved miRNAs, which had potential-binding sites for SERPINC1 3ʹUTR (Figure 2a). Then, the results of RT-qPCR suggested that among these genes, only miR-200c-3p was upregulated in serum of VTE rats (Figure 2b). Moreover, TargetScan database revealed the binding sequence of miR-200c-3p on position 343–349 of SERPINC1 3ʹUTR (Figure 2c). Luciferase reporter assay was conducted to validate the binding of miR-200c-3p on SERPINC1 *in vitro*. The results depicted that miR-200c-3p depletion increased the luciferase activity of SERPINC 3ʹUTR-Wt, but had no influence on the luciferase activity of SERPINC 3ʹUTR-Mut in HEK293 cells (Figure 2d).

(a) TargetScan database predicted five miRNAs potentially harboring conserved binding sites for SERPINC 3ʹUTR. (b) Candidate miRNA expression levels were tested using RT-qPCR in control or thrombotic group. n = 6. (c) Binding sequence of miR-200 c-3p on SERPINC1 3ʹUTR is obtained from TargetScan database. (d) Luciferase reporter assay was conducted to assess the binding ability of miR-200 c-3p and SERPINC1 3ʹUTR. **p < 0.01. miR-NC: miRNA inhibitor negative control; Wt: wild-type; Mut: mutant.

### MiR-200c-3p downregulation suppresses the thrombus formation in VTE rat models

**Figure 3.**
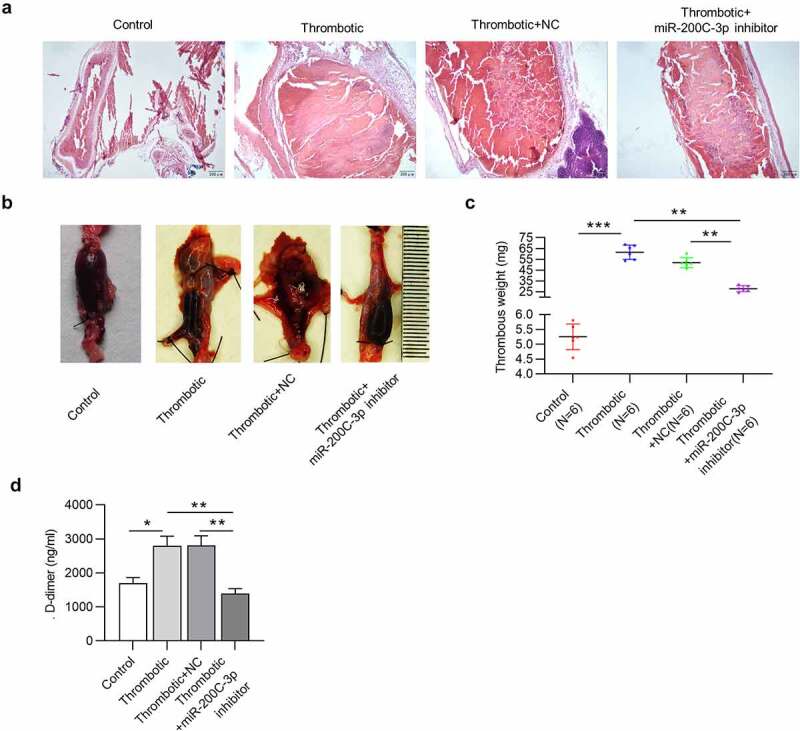
MiR-200c-3p downregulation suppresses the thrombus formation in VTE rat models

Subsequently, we investigated biological function of miR-200c-3p in thrombus formation. Histological examination of IVC containing thrombus by H&E staining confirmed that VTE rats administered with miR-200c-3p inhibitor displayed less intact thrombus in IVC versus rats in thrombotic or thrombotic + NC group (Figure 3a). In addition, the thrombus formation in miR-200c-3p inhibitor-injected VTE rats was reduced, as shown by gross thrombus examination (Figure 3b). Moreover, a remarkable reduction in thrombus weight of VTE rats was observed under miR-200c-3p deficiency (Figure 3c). Furthermore, intravenous injection of miR-200c-3p inhibitor decreased D-dimer level in serum of VTE rats (Figure 3d), which suggested a reduced thrombogenic potential among miR-200c-3p inhibitor-treated VTE rats.

(a) Representative photomicrographs of H&E-stained sections of thrombus with the vessel wall. (b) Representative images of gross IVC with thrombus. (c) Thrombus weight and (d) D-dimer serum level in the thrombotic group administered with miR-200 c-3p inhibitor versus the thrombotic group. n = 6. *p < 0.05, **p < 0.01, ***p < 0.001. NC: miRNA inhibitor negative control.

### MiR-200c-3p negatively regulates SERPINC1 in VTE rat models

**Figure 4.**
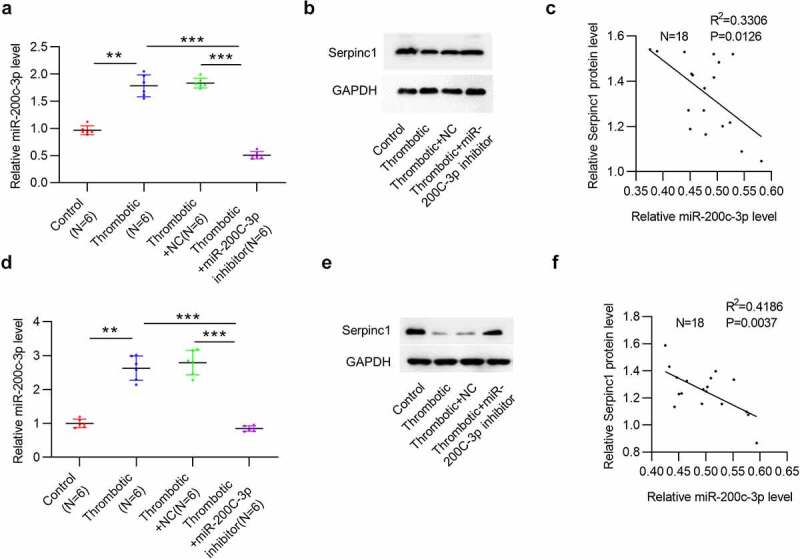
MiR-200c-3p negatively regulates SERPINC1 in VTE rat models

As mentioned in above experiments, SERPINC1 was targeted by miR-200c-3p. We next explored the relationship between miR-200c-3p and SERPINC1 in VTE rat models. RT-qPCR indicated that the level of miR-200c-3p in the serum of VTE rats (n = 6) was reduced after downregulating miR-200c-3p (Figure 4a). Western blotting illustrated that miR-200c-3p depletion rescued the reduced SERPINC1 protein level in serum of VTE rats (Figure 4b). Moreover, linear regression analysis demonstrated that SERPINC1 protein level was negatively correlated with miR-200c-3p level in serum of VTE rats (n = 18) (Figure 4c). Additionally, we found the similar relationship between miR-200c-3p and SERPINC1 in IVC of VTE rats. MiR-200c-3p level in IVC of VTE rats (n = 6) was decreased after administration with miR-200c-3p inhibitor (Figure 4d). MiR-200c-3p downregulation rescued the decreased SERPINC1 protein level in IVC of VTE rats (Figure 4e). Furthermore, a negative correlation between SERPINC1 protein level and miR-200c-3p level was also discovered in IVC of VTE rats (n = 18) (figure 4f).

(a) MiR-200 c-3p level in rat serum in control, thrombotic, thrombotic + NC, thrombotic + miR-200 c-3p inhibitor groups. n = 6. (b) SERPINC1 protein level in rat serum in each group. (c) Scatter plot analysis of the correlation between SERPINC1 protein level and miR-200 c-3p level in thrombotic rat serum. n = 18. (d) MiR-200 c-3p level in rat IVC in control, thrombotic, thrombotic + NC, thrombotic + miR-200 c-3p inhibitor groups. n = 6. (e) SERPINC1 protein level in IVC of rats in each group. (f) Scatter plot analysis of the correlation between SERPINC1 protein level and miR-200 c-3p expression in IVC of thrombotic rats. n = 18. **p < 0.01, ***p < 0.001. NC: miRNA inhibitor negative control.

### SERPINC1 level is negatively correlated with miR-200c-3p level in plasma of VTE patients

**Figure 5.**
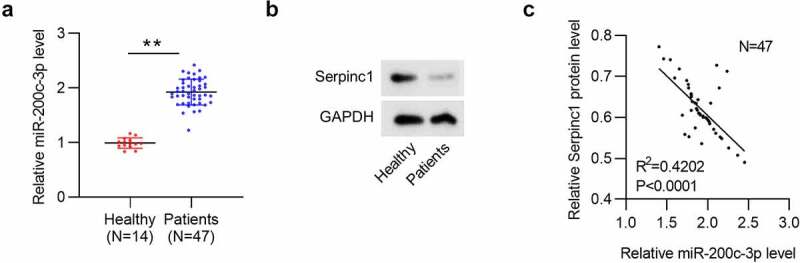
SERPINC1 expression is negatively related to miR-200c-3p level in plasma of VTE patients

Finally, we investigated the relationship between miR-200c-3p and SERPINC1 in VTE development via human studies. Consistent with animal studies, miR-200c-3p level was elevated in plasma of VTE patients (n = 47) versus in healthy controls (n = 14) (Figure 5a). Moreover, SERPINC1 protein level was downregulated in plasma of VTE patients (Figure 5b). In addition, we found the negative correlation between SERPINC1 expression and miR-200c-3p expression in plasma of VTE patients (n = 47) (Figure 5c), suggesting a direct negative regulation of SERPINC1 expression by miR-200c-3p.

(a) Circulatory miR-200 c-3p level and (b) SERPINC1 protein level in plasma of VTE patients (n = 47) and control subjects (n = 14). (c) Scatter plot analysis of the correlation between SERPINC1 protein level and miR-200 c-3p expression in plasma of VTE patients. n = 47. **p < 0.01.**Graphic abstract**: miR-200 c-3p targets SERPINC, the gene for antithrombin, to reduce SERPINC1 translation, ultimately alleviating the aggravation of VTE.

In conclusion, miR-200c-3p targeted SERPINC1, the gene for antithrombin, to reduce the abnormal translation of SERPINC1, thus enhancing the activity of antithrombin, inhibiting the interaction between thrombin and fibrinogen, promoting the conversion of fibrinogen to fibrin, facilitating fibrinolysis, suppressing thrombus formation and ultimately alleviating the aggravation of VTE (Graphic abstract).

## Discussion

In this study, we elucidated the function of miR-200c-3p in thrombosis through both animal models and human samples. We used stasis animal models of VTE that provide a total stasis environment and lead to the most serious vein wall response to thrombosis. Herein, we found that IVC ligation showed the protracted time-dependent influence on the thrombi development inside IVC in VTE rat models. Our study demonstrated that miR-200c-3p downregulation alleviated the thrombi development via targeting SERPINC1, the gene for antithrombin.

SERPINC1 is the short name for serpin peptidase inhibitor, clade C (antithrombin), member 1 [41]. Antithrombin III (ATIII) is a serine protease inhibitor in the coagulation cascade and can be encoded by the gene SERPINC1 [42]. It can profoundly accelerate protease inhibition by interacting with a heparin-like substance on the surface of endothelial cells [42]. Moreover, ATIII increases the production of prostacyclin and then displays powerful anti-inflammatory effects [43,44]. Even minor downregulation of SERPINC1 can increase the risk of thromboembolism [45]. In our report, we found SERPINC1 mRNA and protein levels in VTE rat serum were constantly decreased with thrombus development at 6, 12, 24, and 48 h. Additionally, thrombus weight and SERPINC1 expression showed a negative correlation in serum of VTE rats. These findings suggested SERPINC1 downregulation was associated with thrombus formation.

Previous reports have revealed that miRNAs exert vital effects on venous thrombus formation by modulating target genes. For instance, miR-150 facilitates endothelial progenitor cell angiogenesis and proliferation in deep venous thrombosis through targeting SRC kinase signaling inhibitor 1 (SRCIN1) [46]. Additionally, estradiol-responsive miR-365a-3p binds to the 3ʹUTR of tissue factor (TF) to mediate thrombin generation initiated by TF [47]. One report has revealed that miR-200c-3p promotes the apoptosis of endothelial induced by fluoride via activating Fas pathway [48]. Another report has demonstrated that miR-200c-3p promotes neointimal hyperplasia and transition of endothelial to mesenchymal in artery bypass grafts [49]. Previously, miR-200c-3p, which was downregulated in hibernating black bear plasma to prevent venous thromboembolism during prolonged immobility, was reported to target SERPINC1 [27]. Herein, bioinformatics showed that miR-200c-3p potentially harbored binding site with SERPINC1 3ʹUTR. The binding capacity between miR-200c-3p and SERPINC1 3ʹUTR was further validated through luciferase reporter assay. MiR-200c-3p binds with SERPINC1 3ʹUTR, which leads to SERPINC1 degradation or translational inhibition. Consequently, miR-200c-3p depletion upregulated SERPINC1 expression in VTE. MiR-200c-3p deficiency suppressed the thrombus formation in VTE rat models. In addition, miR-200c-3p was upregulated while SERPINC1 was downregulated and miR-200c-3p expression was correlated with SERPINC1 expression in the IVC and serum of VTE rats and the plasma of VTE patients. Overall, miR-200c-3p downregulation alleviates the thrombi development in VTE via elevating the expression of SERPINC1.

Although novel targets with promising research value were discovered, the present study also has some limitations. Further investigations are required to elucidate the potential downstream signaling pathways of the miR-200c-3p/SERPINC1 axis in VTE in future studies. Furthermore, the upstream molecules that regulate the miR-200c-3p-SERPINC1 network in VTE are still unclear. In addition, even though the functionality of miRNAs in mediating diverse gene expression in various human diseases makes miRNAs an ideal candidate for therapeutic applications [50], there are still several obstacles in miRNA-based therapeutics that need to be overcome, including miRNA stability, off-target effects, renal clearance, the immunogenicity of delivery vehicles or inefficient endocytosis by target cells [51]. Existing studies have demonstrated that one key cause that results in the slow development of miRNA-based therapeutics is ‘too many targets for miRNA effect’ (TMTME) [52]. Consequently, the application of miRNA-based therapeutics in improving the treatment of VTE in the future are also faced with a series of challenges.

## Conclusion

Our research was the first to confirm upregulation of miR-200c-3p as well as downregulation of SERPINC1 in VTE rat models and VTE patients. We innovatively confirmed that miR-200c-3p targets SERPINC, the gene for antithrombin, to reduce SERPINC1 translation and enhance the activity of antithrombin, thereby ameliorating the severity of VTE. Our report might provide a novel insight for VTE clinical treatment.
